# Evaluation of Agraz Consumption on Adipocytokines, Inflammation, and Oxidative Stress Markers in Women with Metabolic Syndrome

**DOI:** 10.3390/nu10111639

**Published:** 2018-11-02

**Authors:** Juliana Espinosa-Moncada, Catalina Marín-Echeverri, Yeisson Galvis-Pérez, Gelmy Ciro-Gómez, Juan C. Aristizábal, Christopher N. Blesso, Maria Luz Fernandez, Jacqueline Barona-Acevedo

**Affiliations:** 1 Food and therapeutic alternatives area, Ophidism Program; School of Microbiology, Universidad de Antioquia UdeA, Calle 70 No. 52-21, Medellín 050010, Colombia; juliana.espinosam@udea.edu.co (J.E.-M.); catalina.marin@udea.edu.co (C.M.-E.); yeisson.galvis@udea.edu.co (Y.G.-P.); gelmy.ciro@udea.edu.co (G.C.-G.); 2 Research Group PHYSIS, School of Nutrition and Dietetics, Universidad de Antioquia UdeA, Calle 70 No. 52-21, Medellín 050010, Colombia; juan.aristizabal@udea.edu.co; 3 Department of Nutritional Sciences, University of Connecticut, Storrs, CT 06269, USA; christopher.blesso@uconn.edu (C.N.B.); maria-luz.fernandez@uconn.edu (M.L.F.)

**Keywords:** *Vaccinium meridionale*, metabolic syndrome, polyphenols, berries, Andean berry, intervention

## Abstract

Metabolic syndrome (MetS) is characterized by increased oxidative stress and a pro-inflammatory state. *Vaccinium meridionale* Swartz (known as “agraz”) is a berry rich in polyphenolic compounds with demonstrated antioxidant activity and anti-inflammatory effects in preclinical studies. The aim of this study was to evaluate the effects of agraz consumption on inflammatory and oxidative stress markers in women with MetS. Forty women with MetS (47 ± 9 years) were randomly assigned to consume daily either 200 mL of agraz nectar or placebo over four weeks in a double-blind, cross-over design study, separated by a 4-week washout period. Metabolic and inflammatory markers in serum and antioxidant/oxidative stress markers in serum and urine were assessed at the end of each period. Serum antioxidant capacity measured by the 2,2-diphenyl-1-picrylhydrazyl (DPPH) method was significantly higher (*p* = 0.028), while urinary 8-hydroxy-2′-deoxyguanosine (8-OHdG) was lower (*p* = 0.041) after agraz consumption, compared to placebo. In conclusion, consumption of agraz during four weeks increased serum antioxidant capacity and decreased a marker of DNA oxidative damage in women with MetS, compared to placebo. These results suggest that agraz consumption may play a protective role in patients with MetS.

## 1. Introduction

The metabolic syndrome (MetS) is a group of risk factors associated with obesity, hypertension, and insulin resistance (IR), which collectively increase the risk for developing type 2 diabetes (T2D) (5-fold) and cardiovascular disease (CVD) (2-fold) [1]. CVD is the leading cause of mortality in the world [2] and in Colombia [3]. The prevalence of MetS in most countries varies between 20% and 30% in the adult population depending on the geographical location and the criteria used for diagnosis [4,5]. In the United States, the general prevalence of MetS is 33%, being significantly higher in women compared to men (35.6% vs. 30.3%, respectively) [6]. In Colombia, there are no reports of MetS prevalence, but some studies have reported a prevalence between 12.3% to 41% in adults from different cities [7,8,9]; with a similar prevalence between men (39.3%) and women (40.7%) in Medellin [10].

MetS includes central obesity, systemic hypertension, insulin resistance, and dyslipidemia (hypertriglyceridemia and low high-density lipoprotein cholesterol (HDL-c)). In addition, MetS is associated with accelerated atherosclerosis in response to chronic inflammation and vascular endothelial dysfunction [4]. This pro-inflammatory environment results from the activation of mediators such as (tumor necrosis factor alpha (TNF-α) and interleukin-6 (IL-6)), plasminogen activator inhibitor-1 (PAI-1), and C-reactive protein (CRP), which act in a variety of organs, but mainly liver and adipose tissue. Importantly, it has been demonstrated that CRP concentrations independently predict the occurrence of future CVD events [11,12]. Increased systemic oxidative stress characterized by increases in free radicals is also observed in MetS [13]. Oxidative stress is associated with endothelial dysfunction and systemic inflammation, and induces oxidative damage to lipids, proteins, and DNA [14]. The effects of oxidative stress can be measured via 8-isoprostane generation, resulting from membrane lipid oxidation of body tissues, and it is associated with the development of CVD [15]. Another marker of oxidative damage affecting DNA is 8-hydroxy-2′-deoxyguanosine (8-OHdG), which is produced from oxidation of guanine and reflects the balance between oxidative damage and repair rate. Urinary 8-OHdG has been proposed as an indicator of oxidative damage [16].

Other important characteristic of MetS is the imbalance in the profile of adipocytokines produced by adipose tissue. Adiponectin has antiatherogenic effects [17], via improving insulin sensitivity, hyperglycemia, and inflammation [18]. Leptin and resistin are also related to obesity, insulin resistance, and inflammation [19,20]. Individuals with MetS generally present high circulating concentrations of resistin and leptin, inducing leptin resistance and a decrease in adiponectin associated with insulin resistance [20].

Currently, the first line treatment for MetS is lifestyle changes such as dietary modifications, increasing physical activity, reduction in alcohol and tobacco consumption, and increased fruit and vegetable consumption [12,21]. When lifestyle changes are not enough, it is necessary to use medications to treat each component of the MetS; however, this may come with unwanted side effects of polymedication and contributes to the high costs of healthcare systems, representing a public health problem that needs to be ameliorated [22]. Therefore, it is crucial to identify strategies targeting these multiple risk factors associated with MetS to improve population health and to mitigate secondary effects, which is the goal of the World Health Organization in order to reduce the incidence and mortality from CVD and T2D [23].

Regular consumption of fruits and vegetables has been associated with a lower prevalence of CVD and mortality [24,25], the benefits have been attributed in part to the content of polyphenolic compounds, such as anthocyanins, present in these foods. Different studies have shown that polyphenols exhibit anti-inflammatory and antioxidant effects, among others [26,27]. Berries in particular, are fruits rich in these compounds and their chronic consumption has demonstrated multiple positive effects on metabolism [28,29,30].

The berry *Vaccinium meridionale* Swartz is a native plant from the Andean region of South America, growing on mountain hillsides between 2200 and 3400 m above sea level [31]. In Colombia, this fruit is called “agraz” and it has generated significant interest in the food and pharmaceutical industries due to its antioxidant, anti-inflammatory, and anticarcinogenic potential [32,33]. Thus, it might have the ability to modulate MetS risk factors [34]. Recent studies reported cardioprotective effects of an extract from this berry (*V. meridionale*) in rodents [35,36]. However, there is very limited information about its potential human health benefits. Therefore, the aim of this study was to evaluate the effects of agraz consumption on inflammatory and oxidative stress markers in women with MetS. We hypothesized that compared to placebo, agraz would decrease inflammation/oxidative stress markers in these volunteers. 

## 2. Materials and Methods

### 2.1. Experimental Design

Forty women (*n* = 40; 28–66 years old) classified with MetS, according to the revised American Diabetes Association-National Cholesterol Education Program (AHA-NCEP)-ATPIII definition [37], were recruited to participate in this study. According to this definition of MetS, participants must have 3 out of 5 of the following characteristics: waist circumference (WC) ≥ 88 cm, blood pressure (BP) ≥ 130/85 mm Hg, plasma triglycerides (TG) ≥ 150 mg/dL, HDL-c < 50 mg/dL, and fasting plasma glucose ≥ 100 mg/dL. Since there are no previous studies in people consuming this fruit, the sample size estimation and the time of supplementation were based on data from two previous studies following a similar protocol as this study, in which blood pressure, plasma triglyceride, low-density lipoprotein cholesterol (LDL-c), oxidative stress, and inflammatory markers were reduced significantly after grape supplementation (a fruit rich in polyphenols), compared to placebo [38,39]. The exclusion criteria were kidney disease, CVD, TG ≥ 500 mg/dL, fasting blood glucose ≥ 126 mg/dL or having diabetes, LDL-c ≥ 190 mg/dL, and BP > 140/90 mm Hg. In addition, individuals were excluded if they were taking lipid-lowering, hypoglycemic and anti-hypertensive medications, acetylsalicylic acid, warfarin or other anticoagulants, ibuprofen, clopidogrel, naproxen, dipyridamole, and any other nonsteroidal anti-inflammatory drug, as well as undergoing hormone replacement therapy, consuming >20 g per day of alcohol, cigarette smoking, being pregnant or planning to become pregnant, being a high performance athlete, and/or consumption of supplements or nutraceuticals.

The Human Bioethical Committee of the University Research Headquarters, University of Antioquia, approved the study with the Act No. 15-35-558-02. All participants signed the informed consent before entering the study. The first participant entering the study was randomly assigned to consume either agraz or placebo; the rest of the participants were assigned in such an order that each consumption period began with the same number of participants, in a double-blinded crossover experimental design over 12 weeks. The placebo was designed to match the agraz in terms of look, feel, taste, and macronutrients, but without polyphenols. The composition of agraz nectar and placebo is presented in Table 1 and it has been previously described [40]. Following 4 weeks of consuming agraz or placebo, participants underwent a 4-week washout period and were allocated to the alternate treatment for the next 4 weeks. The daily dose of agraz was calculated taking into account the total content of phenols in 200 g of fresh fruit. The corresponding amount of lyophilized agraz and placebo was reconstituted in 200 mL of drinkable water. Participants were asked to abstain from consuming polyphenol-rich foods, including tea, berries, grapes, and wine, during the whole study, in addition to maintaining their level and type of physical activity and usual diet throughout the study. To monitor the adherence to the study, participants completed weekly questionnaires asking about timing and days of consumption of the product and if any prohibited food had been consumed, for which they needed an adherence greater than 80% to continue in the study. Additionally, participants completed physical activity and dietary registries at the beginning and last week of each consumption period (agraz and placebo). The adherence and physical activity registries and food frequency questionnaire were adapted from Barona et al. [39] and Monsalve et al. [41], respectively.

### 2.2. Anthropometrics: Body Weight, Height, Body Mass Index (BMI), and Waist Circumference (WC)

Body weight was measured to the nearest 0.1 kg on a calibrated digital scale (Seca 813, Seca, Chino, CA, USA). Height was measured to the nearest 0.1 cm using a portable stadiometer (Seca 213, Seca, Chino, CA, USA). BMI (kg/m^2^) was calculated to classify participants [42]. WC was measured at the upper border of the iliac crest to the nearest 0.1 cm using a non-stretching body measuring tape over the skin (Lufkin W606PM, Crescent Tools, MD, USA), at the beginning and end of each period.

### 2.3. Blood Pressure (BP)

Systolic and diastolic BP were measured in the left arm at the level of the heart after at least 5 min of resting in sitting position and using an automated BP monitor (Omron Healthcare Inc., Hoffman Estates, IL, USA). At least two measurements were made separated by 1 min and the mean value was used.

### 2.4. Blood and Urine Collection

After a 12-h overnight fast, blood was drawn from the antecubital vein using dry tubes (Vacutainer^®^, Franklin Lakes, NJ, USA) which were allowed to stand for 30 min and centrifuged at 2000× *g* for 10 min. Serum was aliquoted and frozen at −70 °C until analysis. Urine samples were collected for 24 h without preservatives and kept in refrigeration during collection. The urine was aliquoted and stored at −70 °C until analysis.

### 2.5. Blood Lipids, Glucose and Insulin, and Urinary Creatinine

Fasting blood lipids (total cholesterol, HDL-c, non-HDL-c, and TG) and glucose, and urinary creatinine were measured in an automatic analyzer (SIEMENS, Washington, DC, USA). LDL-c concentration was calculated using the Friedewald formula [43]. For the determination of insulin, a sandwich immunoassay was employed with direct chemiluminescence technology (SIEMENS, Washington, DC, USA) in an automated analyzer. Atherogenic indexes of plasma were calculated based on the literature [44,45].

### 2.6. Index of Insulin Resistance

The Homeostatic Model Assessment for Insulin Resistance (HOMA-IR) was calculated using the free software HOMA calculator V2.2.3 (Diabetes Trial Unit, University of Oxford, Headington, Oxford, UK). The QUICKI index was calculated using the formula Quicki = 1/[log insulin (mUI/L) + log glucose in fasting (mg/dL)] [46].

### 2.7. Inflammation Markers and Adipocytokines

Levels of high sensitive CRP (hs-CRP) were measured using the turbidimetric immunoassay technique (SIEMENS, Washington, DC, USA). Serum adiponectin, leptin, and resistin were measured using the human adipocyte magnetic panel kit (Millipore Sigma, Burlington, MA, USA), using Luminex xMAP^®^ technology (Millipore Sigma) and following the manufacturer’s instructions.

### 2.8. Total Phenols Concentration and Antioxidant Capacity

The serum of the patients was deproteinized by the method of Serafini et al. [47] with modifications. Total phenols were determined in deproteinized serum using the methodology described by Singleton V. L. et al. by the Folin–Ciocalteu method [48], the values were calculated from a calibration curve with gallic acid (GA) and the results were expressed as mg of GA equivalents (GAE)/L. Antioxidant capacity was determined by the free radical method 2,2-Diphenyl-1-Picrylhydrazyl (DPPH). DPPH scavenging activity was measured using a modification of the method described by Chrzczanowicz et al. [49], using deproteinized serum and control (mix of the reagent to deproteinize without serum). The scavenging effect (Sc%) of DPPH was calculated using the following formula. Sc% = (1 − (A517 serum sample/A517 control)) × 100.

### 2.9. Oxidative Stress Markers

The determination of thiobarbituric acid reactive substances (TBARS) was carried out with the TBARS (Trichloroacetic acid method) Assay Kit (Cayman Chemical, Ann Arbor, MI, USA) using a colorimetric standard and following manufacturer’s recommendations. The measurement of F2-isoprostanes in 24-h urine was performed with the OxiSelect™ 8-iso-prostaglandin F2α ELISA kit (Cell Biolabs, Inc., San Diego, CA, USA). A competitive Elisa kit was used to measure 8-OHdG in 24-h urine (Abcam, Cambridge, MA, USA). Urinary creatinine was used as a control for the 24-h urine collection and to normalize the values of urinary F2-isoprostanes and 8-OHdG, which are presented as ng/mg creatinine.

### 2.10. Statistical Analysis

The Shapiro–Wilk normality test was used to determine the distribution of the variables. For those showing a normal distribution, a *t*-test was performed for paired samples and for non-normally distributed variables, the Wilcoxon test was used to analyze the differences between the periods of agraz and placebo. Pearson’s and Spearman’s correlation coefficients were used to evaluate associations between quantitative variables measured in agraz or placebo periods according to their distribution. A *p*-value ≤ 0.05 was considered significant. These analyses were carried out using SPSS software version 24.0 for Windows (IBM Corporation, Armonk, NY, USA, 2016). Results are presented as median (interquartile range—IQR) and mean ± standard deviation-SD.

## 3. Results

Participants maintained their usual diet and physical activity, and their body weight was constant throughout the study. Adherence to the study was greater than 90%. Thus, the obtained results were not associated with weight loss, changes in the composition (quantity and quality) of the diet, or changes in energy expenditure.

### 3.1. MetS Criteria and Participant Characteristics

**Figure 1:**
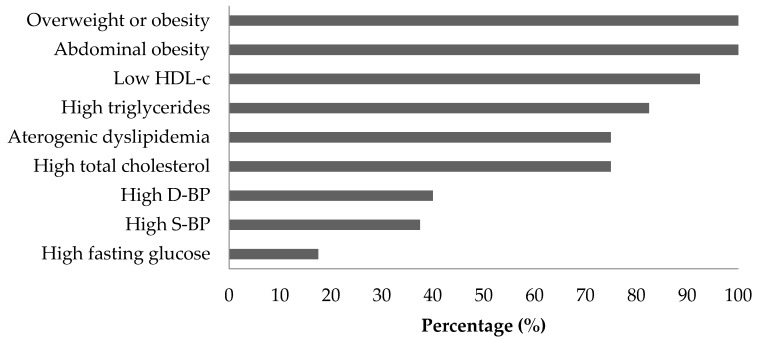
Baseline clinical and anthropometric characteristics of women with metabolic syndrome [according to ATP-III guidelines (37)]. HDL-c: high-density lipoprotein cholesterol. D-BP: diastolic blood pressure. S-BP: systolic blood.

The mean age of participants was 47.3 ± 9.4 years. All women were overweight or obese (BMI 29.4 (3.8) Kg/m^2^) and had abdominal obesity (99.4 (10.2) cm). Low HDL-c (42 ± 6.4 mg/dL) was also present in most women (92.5%). High fasting glucose was the least frequent parameter in the population (17.5%) (Figure 1). At the beginning of the study, the percentage of participants having three, four, or five MetS criteria was 62.5%, 35%, and 2.5%, respectively. At the end of the agraz consumption period, 22.5% of the women had only one or two MetS criteria, that is, they no longer had MetS. Following agraz consumption, it was observed that TG, total cholesterol, LDL-c, and non-HDL-c tended to be lower than placebo, but it was not statistically significant (Table 2). Regarding physical activity and diet, no statistically significant differences were found between the periods (data not shown).

### 3.2. Antioxidant Capacity and Oxidative Stress Markers

Antioxidant capacity measured by DPPH was higher after agraz consumption compared to placebo (+2.08 ± 5.75, *p* = 0.028). The concentration of 8-OHdG oxidation marker was significantly lower after consuming agraz (−0.27 ± 0.72, *p* = 0.041) (Table 3). No statistically significant differences were found between periods in total phenols, TBARS or F2-isoprostanes.

### 3.3. Insulin Resistance and Inflammatory Markers

**Figure 2:**
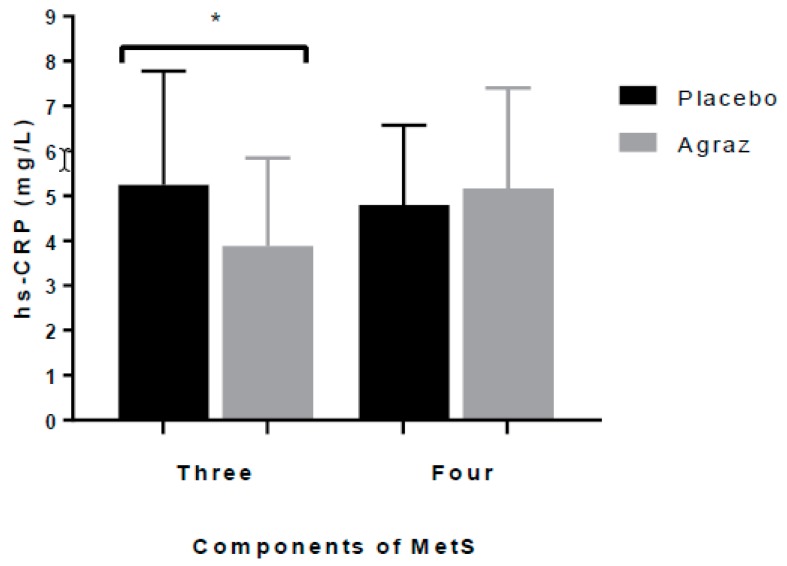
High sensitive C-reactive Protein (hs-CRP) levels in patients who presented three and four+ metabolic syndrome (MetS) parameters after placebo and agraz consumption. Values are presented as mean ± standard deviation. * Significance *p* ≤ 0.05.

The concentration of markers of insulin resistance and inflammation were not different after agraz compared to placebo period (Table 4). However, the median of hs-CRP was 21.9% lower (although no significant, *p* = 0.103) after agraz consumption compared to the median of placebo. Additionally, compared to placebo, a statistically significant decrease in hs-CRP values was found after agraz period in women who met three MetS criteria (−1.06 ± 2.52 mg/L, *p* = 0.011) but not in those with four or more criteria at the beginning of the study (0.36 ± 1.47 mg/L, *p* = 0.36) (Figure 2). No statistically significant differences were found in the concentration of other inflammatory markers and adipocytokines.

### 3.4. Correlations

**Figure 3:**
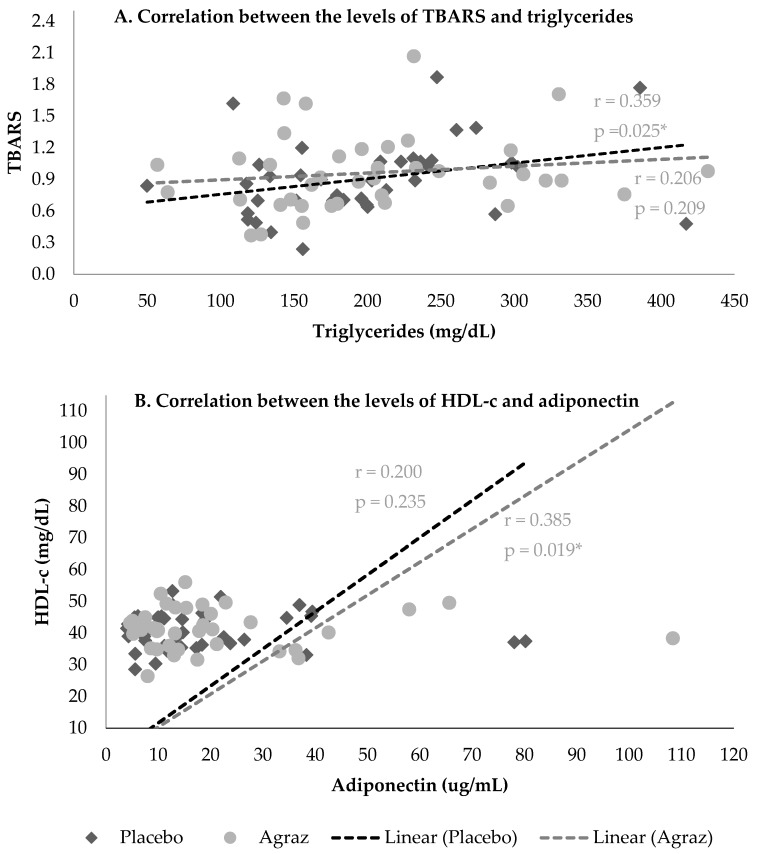
Spearman correlations between the concentrations of Triglycerides and TBARS (**A**) and HDL-c and adiponectin (**B**) after agraz and placebo consumption in women with MetS. * *p* ≤ 0.05 is considered significant. r: correlation coefficient.

A positive correlation was found between plasma TG levels and TBARS in the period of placebo consumption (r = 0.355, *p* = 0.025), but this correlation lost significance after agraz consumption (r = 0.206; *p* = 0.209). Furthermore, after agraz consumption there was a positive correlation between the levels of HDL-c and adiponectin (r = 0.385; *p* = 0.019). Interestingly, this correlation was not observed after placebo period (Figure 3).

## 4. Discussion

The increased consumption of fruits and vegetables has shown to improve different parameters of MetS, which is attributed in large part to the polyphenolic compounds present in these foods with antioxidant, anti-inflammatory, and anticancer activities (reviewed previously [50]). In our study, the consumption of agraz nectar for four weeks, compared to placebo, significantly decreased the oxidative marker 8-OHdG in the participants. These results may be associated with the significant increase in serum antioxidant capacity measured by the DPPH radical after the agraz period. In contrast, McAnulty et al. (2011) found no significant differences in the DNA oxidation assay (8-OHdG) after supplementation with blueberries and placebo plus exercise (stress condition), but they did find a significant decrease in F2-isoprostanes in the group receiving blueberry treatment [51].

Results reported after supplementation with fruits rich in polyphenols are controversial. Novotny et al. (2015) examined the effects of consuming cranberry juice low in calories plus a diet modification during eight weeks in men and women with obesity and cardiovascular risk. Compared to the control group (only diet modification), plasma levels of TG were lower in patients who consumed juice, especially in patients who had higher TG concentrations at the beginning of treatment [52]. In addition, plasma hs-CRP concentration, fasting glucose, and diastolic blood pressure were lower in the group consuming juice compared to the control group. Consumption of this cranberry juice also had a beneficial effect on HOMA for participants with high baseline values for this marker. These findings suggested that cranberry juice could improve some risk factors associated with CVD in overweight adults in addition to the effects of the diet alone (control group) [52]. In contrast, Kolehmainen et al. (2012) measured the impact of *V. myrtillus* (bilberry) consumption during eight weeks, on inflammation and gene expression profile in peripheral blood mononuclear cells isolated from 27 subjects with MetS. Participants were randomly assigned to a diet rich in *V. myrtillus* supplied as 200 g of puree and 40 g of dried fruit or to the control diet group. No significant differences were found between the two groups in terms of body weight, blood pressure, plasma glucose concentration, or measures of lipid metabolism; whereas in the group undergoing treatment with *V. myrtillus*, a tendency to decrease the concentration of hs-CRP and some proinflammatory cytokines was observed. The authors concluded that regular *V. myrtillus* consumption can reduce inflammation, and thus, decrease long-term cardiometabolic risk [53]. Similarly, we found lower values of TG, total cholesterol, LDL-c, and hs-CRP after consuming agraz compared to placebo, but these differences were not significant. It would be necessary to explore a higher dose and/or increase the time of supplementation to evaluate whether significant differences would be observed in these markers.

Previous studies have shown that hs-CRP concentrations are related to the number of MetS components [54,55], with higher levels of hs-CRP as MetS components increase. After analyzing women by components of MetS, values of hs-CRP decreased significantly after agraz consumption, compared to placebo in women having three MetS parameters at baseline, but not in those with four or five parameters; suggesting a more preventive effect of this fruit in those with less severe forms of MetS. Several studies have found an association between consumption of cranberry and blueberries and a decrease on hs-CRP and other inflammation markers [56,57]. This could be explained by the ability of polyphenols to modulate the nuclear transcription factor kappa B (NFκB) pathway, a critical transcription factor implicated in the regulation of oxidative stress and the inflammatory response [58]. Karlsen et al. (2010), after supplementing with *V. myrtillus*, compared to control, reported a reduction in hs-CRP and some pro-inflammatory cytokines by modulation of NFκB [59]. Conversely, Soltani et al. (2016) found that compared to placebo, an encapsulated extract rich in anthocyanins from *V. arctostaphylos* L. consumed during four weeks had no effect on hs-CRP levels, but it significantly reduced total cholesterol and TG concentrations [60].

The relationship between high TG, inflammation, and increased systemic oxidative stress is well known [61]. In our study, we observed a moderate positive correlation between the concentration of TG and concentration of TBARS during the placebo period, but this correlation was not significant after consuming agraz. These results suggest that agraz consumption may help to decrease the relationship between high TG and elevated markers of lipid peroxidation. An intervention with pomegranate juice for six weeks showed that consumption of this polyphenol-rich fruit managed to decrease the concentration of arachidonic acid and levels of TBARS in blood [62]. In addition, a study with juice and smoothies rich in anthocyanins during two weeks showed a significant decrease, compared to placebo, in different markers of oxidative stress, including TBARS [63]. Although there are no other studies reporting TBARS measurement after agraz supplementation in humans, previous in vitro studies have shown the ability of agraz to inhibit lipid peroxidation in corn oils [64]. The presence of a high concentration of anthocyanins in agraz could be associated with this antioxidant activity [31,65].

The experimental evidence indicates that adiponectin has antiatherogenic, anti-inflammatory, and antidiabetic properties [66,67,68]. In addition, previous studies have shown a negative correlation between adiponectin and TG concentrations and a positive correlation with HDL-c in young patients, demonstrating an association between this adipocytokine and dyslipidemia [69]. Interestingly, we found a moderate positive correlation between HDL-c and adiponectin, but only in the period of agraz consumption. Some investigations suggest that antioxidants can regulate adiponectin expression through a reduction of oxidative stress [70,71]. Diets that increase antioxidant capacity have been reported to also increase plasma adiponectin. This might be related to an adiponectin-mediated route through which antioxidant-rich foods can exert beneficial effects against inflammation and CVD [72]. Indeed, adiponectin expression seems to be increased under stimuli of several dietary polyphenols [73,74,75]. Additionally, it has been reported that anthocyanin supplementation improves HDL-c and enhances cellular cholesterol efflux to serum [76]. Reports of interventions with fruits rich in polyphenolic compounds have shown a positive effect in adipocytokines, especially adiponectin [77,78]. Simão et al. (2013) found that consumption of cranberry juice significantly increased serum adiponectin concentrations compared to baseline and placebo in patients with MetS [79]. We found an increase in almost 1 µg/mL in adiponectin after agraz consumption, although this was not significant, it has been reported that an increase in 1 µg/mL is associated with a decrease in 3% in CVD risk [80]. Additionally, Kowalska et al. (2015) found that after administering a lyophilisate of cranberry (*Oxycoccus quadripetalus*) to cell culture of 3T3-L1 adipocytes, there was an increase in the expression of the adiponectin gene and protein secretion in a dose-dependent manner [81]. They finally concluded that berries could be considered as a source of bioactive factors with the ability to modulate gene expression and inhibiting abnormal production of molecules by adipose tissue.

In summary, our results suggest that chronic consumption of agraz in women with MetS decreases oxidative damage in DNA, possibly through a mechanism dependent on the increase in serum antioxidant capacity promoted by agraz. In addition, in women with three components of MetS, the consumption of this fruit reduced hs-CRP levels compared to placebo, but not in women with four or more components, suggesting a protective role in inflammation in less severe cases of MetS. Finally, important correlations with markers involved in lipid peroxidation and antiatherogenic molecules were observed after agraz consumption; supporting the potential of this Colombian fruit to modulate CVD risk factors.

This is one of the first studies evaluating the effects of this fruit in humans. Although the current sample size may have prevented us from finding other significant results, the dose and appearance (nectar) used represented the consumption of agraz in a usual Colombian diet. Due to the astringent flavor of agraz, a more concentrated nectar was not palatable for volunteers. However, more studies are needed to evaluate higher doses and time of supplementation in people at high risk for CVD.

## Figures and Tables

**Table 1:** Phytochemical composition and nutrient analysis of the freeze-dried agraz, nectar, and placebo.

Nutrient/Compound	Freeze-Dried Agraz	Nectar (Reconstituted from Freeze-Dried Agraz in 200 mL)	Placebo (200 mL)
Macronutrients			
Calories per dose (Kcal)	ND	26 ± 0.18	21.86 ± 0.21
Total carbohydrate (%)	78.85 ± 0.19	2.08 ± 0.19	2.50 ± 0.19
Protein (%)	1.39 ± 0.25	0.05 ± 0.01	0.21 ±0.05
Phytochemicals			
Total Phenols (mg GAE/g of freeze-dried or L)	139.29 ± 5.69	1027.97 ± 41.99	31.91 ± 3.15
Anthocyanins (mg CE/g) ^a^	4.66	ND	ND
Anthocyanins (mg/L) ^b^	ND	75.65	ND

ND, non-determined. GAE, gallic acid equivalents; CE, cyanidin equivalents. ^a^ Freeze-dried agraz anthocyanins were analyzed using high performance liquid chromatography diode array detection (HPLC-DAD). ^b^ Nectar anthocyanins were measured by differential pH.

**Table 2:** Changes in anthropometric and biochemical variables in women with metabolic syndrome after consuming agraz compared to placebo.

Variables	Placebo		Agraz		Δ (Agraz-Placebo)	*p*
	*n*	Mean or Median ± SD or IQR (p25-p75)	*n*	Mean or Median ± SD or IQR (p25-p75)	Mean or Median ± SD or IQR (p25-p75)	
**Weight (Kg) ^a^**	40	76.6 ± 11.6	40	76.6 ± 11.6	−0.05 ± 0.9	0.756
**Body Mass Index (Kg/mt^2^) ^b^**	40	29.8 (4.26)	40	29.9 (3.87)	−0.11 (0.64)	0.975
**Waist circumference (cm) ^b^**	40	98.7 (9.43)	40	98.5 (10.23)	−0.2 (3.87)	0.185
**Systolic blood pressure (mm Hg) ^a^**	40	115.4 ± 12.5	40	116.0 ± 11.8	0.5 ± 8.6	0.692
**Diastolic blood pressure (mm Hg) ^a^**	40	74.8 ± 8.0	40	75.1 ± 9.5	0.3 ± 5.2	0.73
**Fasting glucose (mg/dL) ^a^**	40	96.8 ± 8.1	40	95.9 ± 8.0	−0.9 ± 0.3	0.291
**Triglycerides (mg/dL) ^b^**	39	197.61 (104.59)	39	193.82 (105.32)	−9.79 (110.53)	0.759
**Total cholesterol (mg/dL) ^a^**	40	219.6 ± 43.3	40	216.1 ± 45.1	−3.5 ± 38.7	0.574
**HDL cholesterol (mg/dL) ^a^**	40	41.6 ± 6.4	40	41.7 ± 6.8	0.9 ± 4.4	0.905
**LDL cholesterol (mg/dL) ^a^**	36	137.9 ± 39.3	36	132.2 ± 42.1	−5.7 ± 35.1	0.339
**Non-HDL cholesterol (mg/dL) ^a^**	39	177.3 ± 42.3	39	174.5 ± 44.3	−2.8 ± 38.2	0.649
**TG/HDL-c index ^b^**	39	4.45 (2.81)	39	4.84 (3.55)	−0.23 (3.57)	0.606
**TC/HDL-c index ^a^**	40	5.35 ± 1.2	40	5.27 ± 1.2	−0.09 ± 0.9	0.565
**LDL-c/HDL-c index ^a^**	36	3.29 ± 0.96	36	3.17 ± 0.95	−0.12 ± 0.8	0.357

^a^ Paired T; ^b^ Wilcoxon; SD: Standard deviation; IQR: Interquartile range; Significance *p* ≤ 0.05. TG: triglycerides. TC: total cholesterol. HDL: high-density lipoprotein cholesterol. LDL: Low-density lipoprotein cholesterol.

**Table 3:** Changes in antioxidant capacity and oxidation markers in women with metabolic syndrome after agraz consumption compared to placebo.

Variables	Placebo		Agraz		Δ (Agraz-Placebo)	*p*
	*n*	Mean or Median ± SD or IQR (p25-p75)	*n*	Mean or Median ± SD or IQR (p25-p75)	Mean or Median ± SD or IQR (p25-p75)	
**DPPH (% Scavenging effect) ^a^**	40	10.55 ± 6.19	40	12.63 ± 7.47	2.08 ± 5.75	0.028 *
**Total phenols mgGA/L ^b^**	40	297.29 (57.29)	40	331.88 (56.04)	7.92 (72.70)	0.279
**TBARS ^b^**	40	0.89 (0.39)	40	0.92 (0.45)	0.04 (0.42)	0.402
**F2-Isoprostanes (ng/mg creatinine) ^b^**	35	2.86 (3.97)	35	3.03 (3.41)	0.11 (3.39)	0.863
**OHdG (ng/mg creatinine) ^a^**	35	1.97 ± 0.66	35	1.66 ± 0.5	−0.27 ± 0.72	0.041 *

^a^ Paired T; ^b^ Wilcoxon; SD: Standard deviation; IQR: Interquartile range; * Significance *p* ≤ 0.05. DPPH: 2,2-Diphenyl-1-Picrylhydrazyl; TBARS: Thiobarbituric acid reactive substances; 8-OHdG: 8-hydroxy-2′-deoxyguanosine.

**Table 4:** Changes in markers of insulin resistance and inflammation in women with metabolic syndrome after agraz consumption compared to placebo.

Variables	Placebo		Agraz		Δ (Agraz-Placebo)	*p*
	*n*	Mean or Median ± SD or IQR (p25-p75)	*n*	Mean or Median ± SD or IQR (p25-p75)	Mean or Median ± SD or IQR (p25-p75)	
**Insulin (mUI/L) ^b^**	39	16.34 (13.03)	39	15.0 (14.01)	0.26 (4.91)	0.922
**HOMA 2 index ^b^**	38	2.33 (1.83)	40	2.21 (1.8)	0.02 (0.66)	0.577
**QUICKI index ^a^**	39	0.314 ± 0.024	39	0.315 ± 0.024	0.0 ± 0.012	0.714
**hs-CRP (mg/L) ^b^**	37	4.8 (2.81)	37	3.75 (2.80)	−0.54 (2.5)	0.103
**Adiponectin (ug/mL) ^b^**	37	12.75 (14.53)	37	13.23 (11.38)	0.89 (4.43)	0.225
**Resistin (ng/mL) ^a^**	38	31.96 ± 7.77	38	33.84 ± 10.1	1.88 ± 9.45	0.229
**Leptin (ng/mL) ^a^**	38	3.58 ± 1.52	38	3.58 ± 1.66	0.025 ± 0.76	0.986

^a^ Paired T; ^b^ Wilcoxon; SD: Standard deviation; IQR: Interquartile range; Significance *p* ≤ 0.05. hs-CRP: high-sensitive C-reactive protein.
